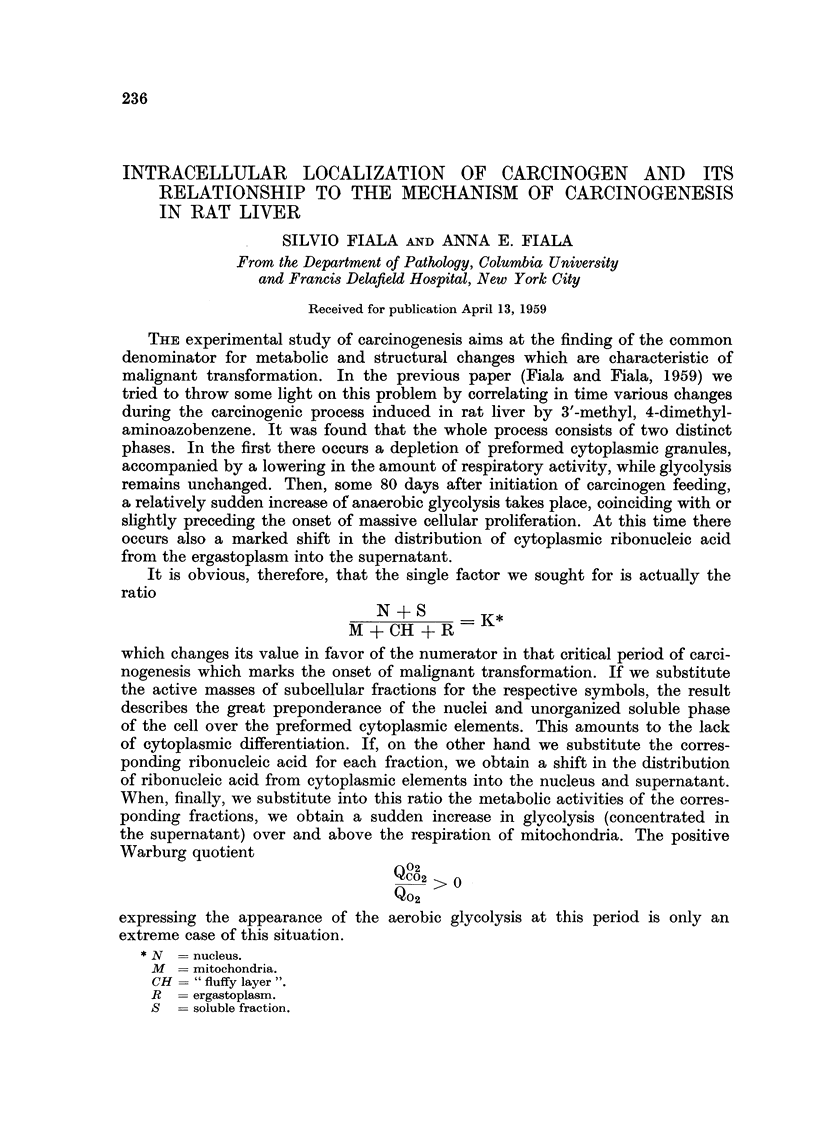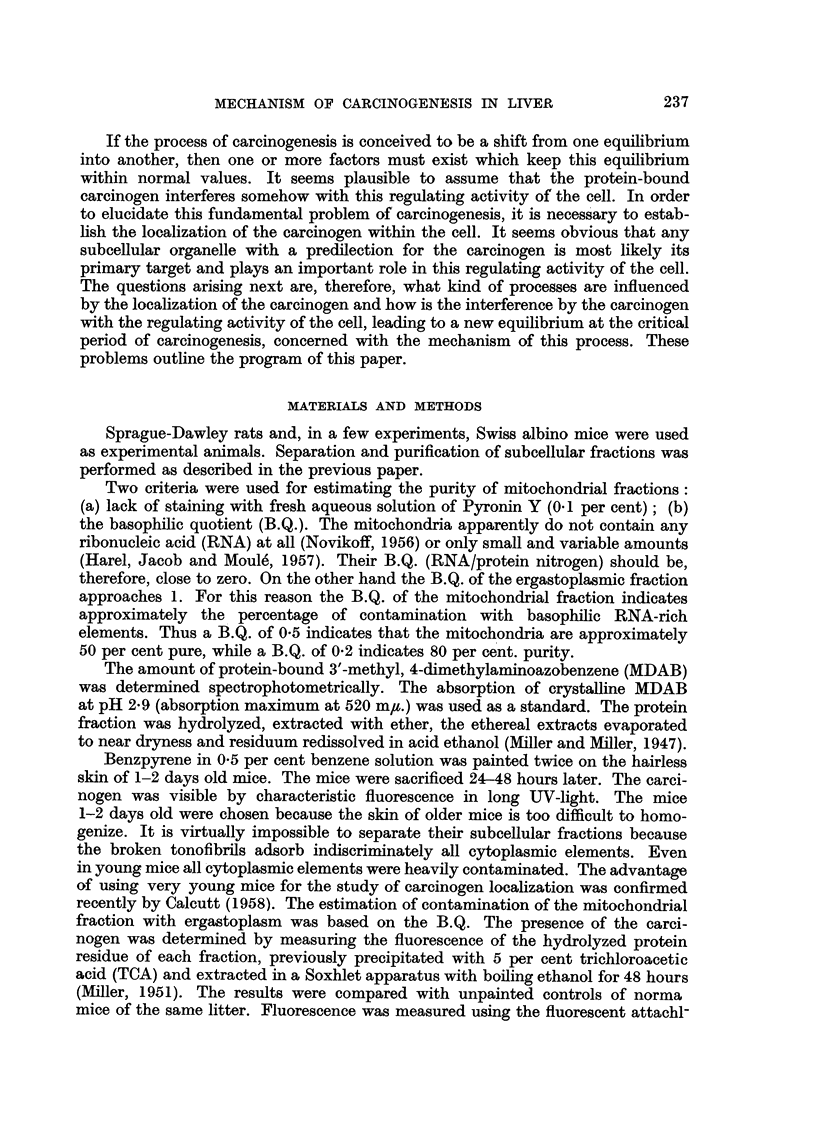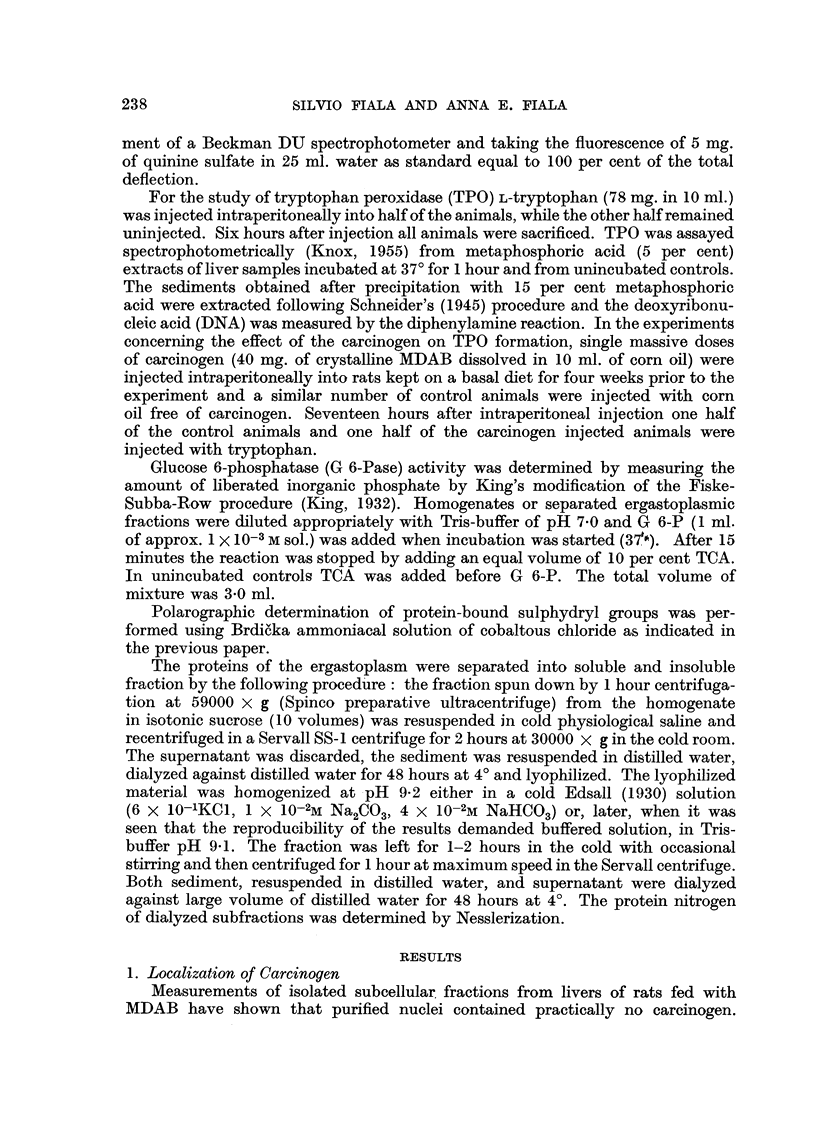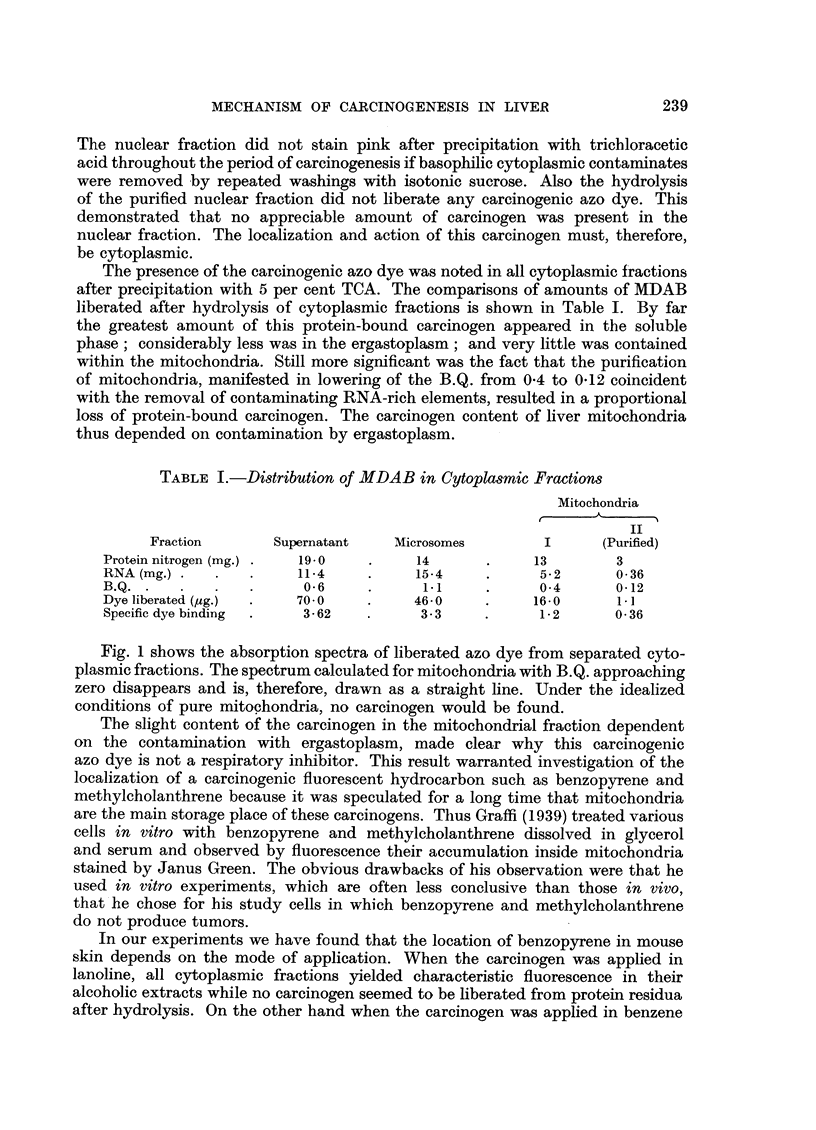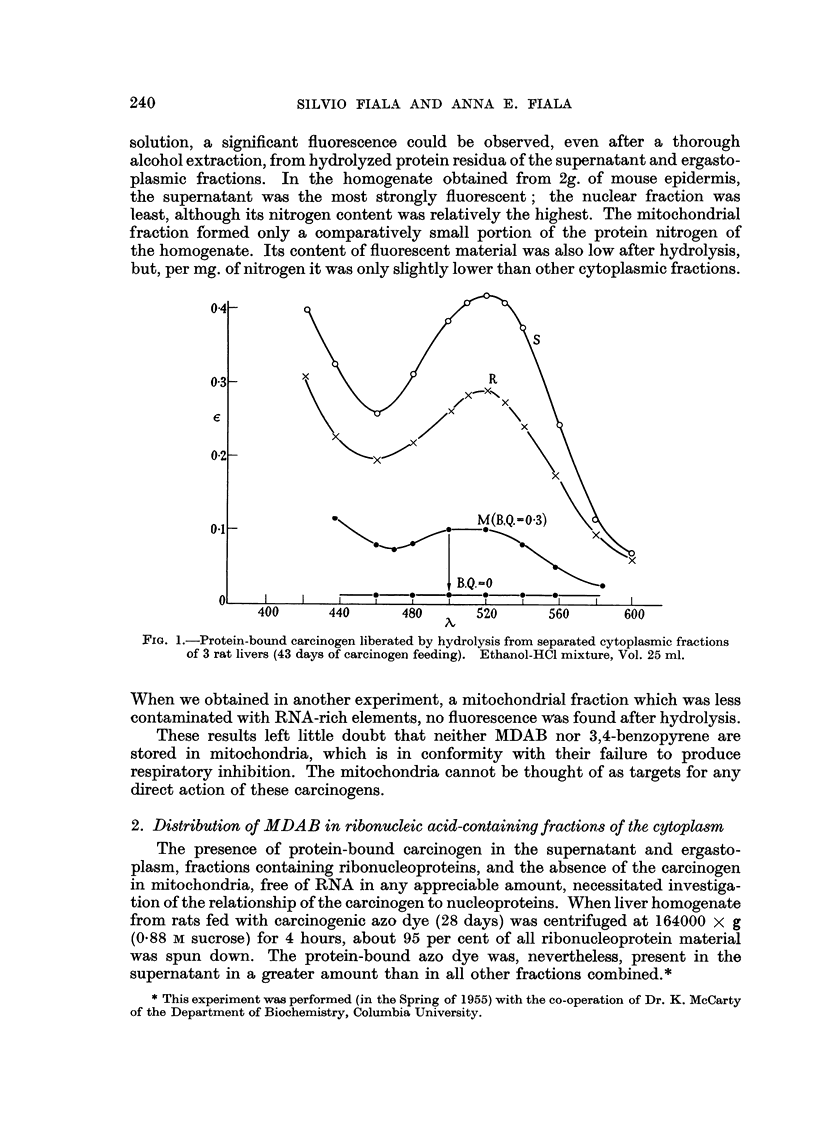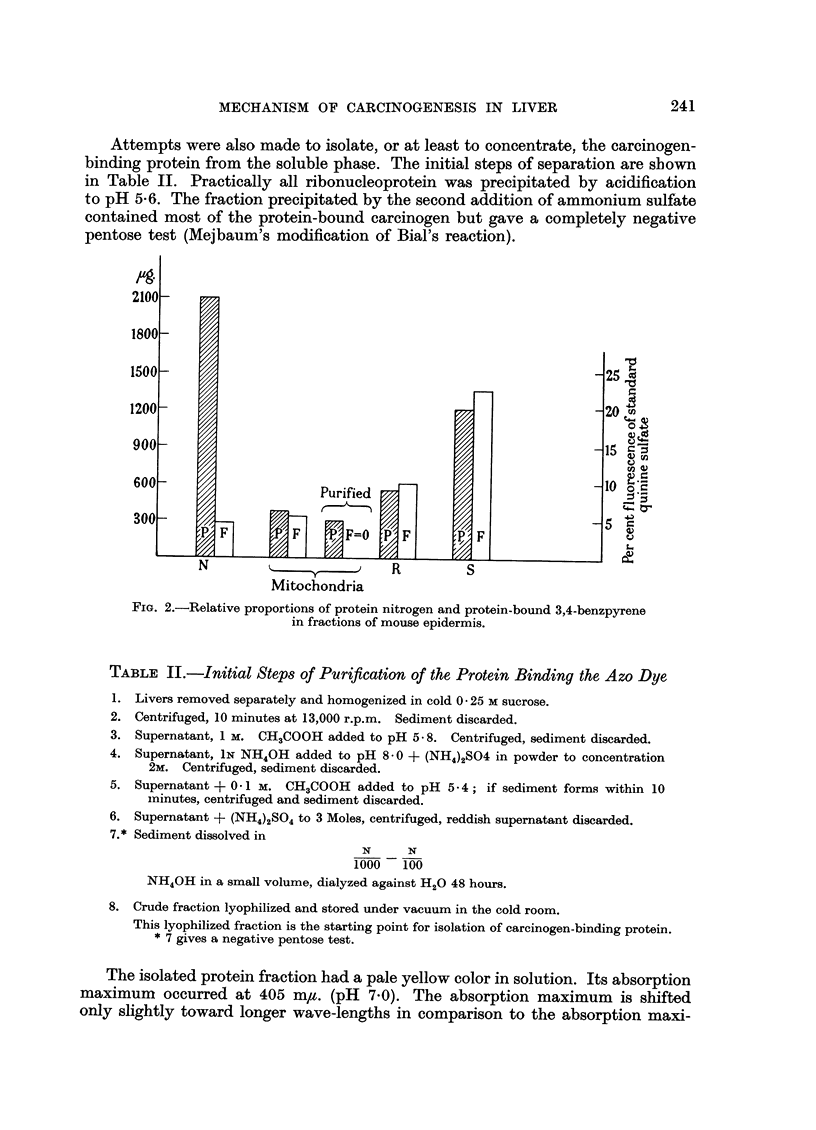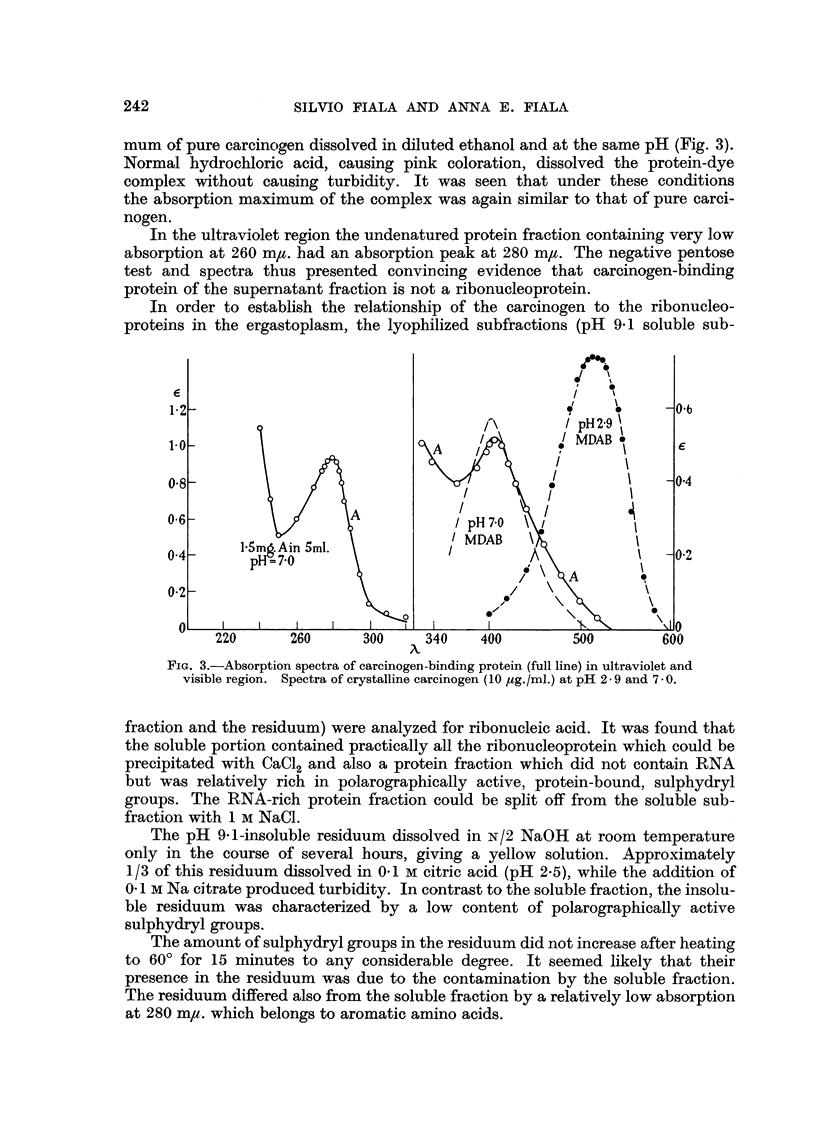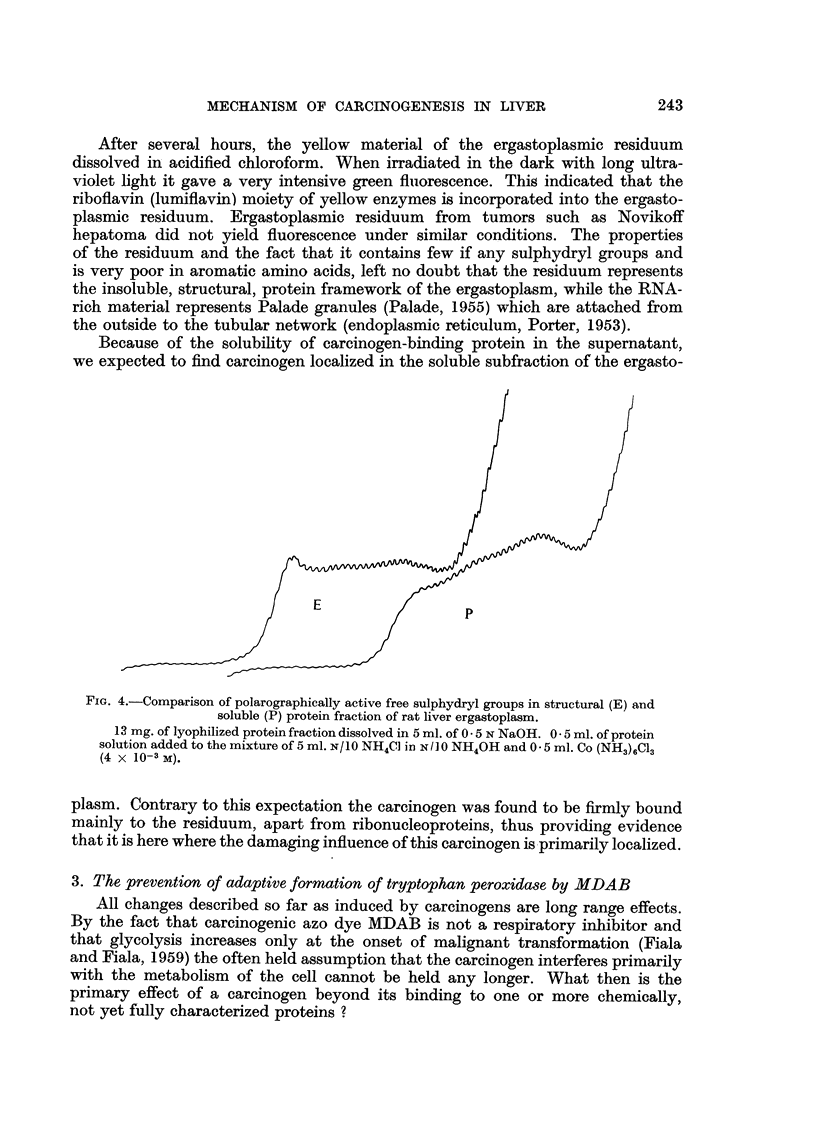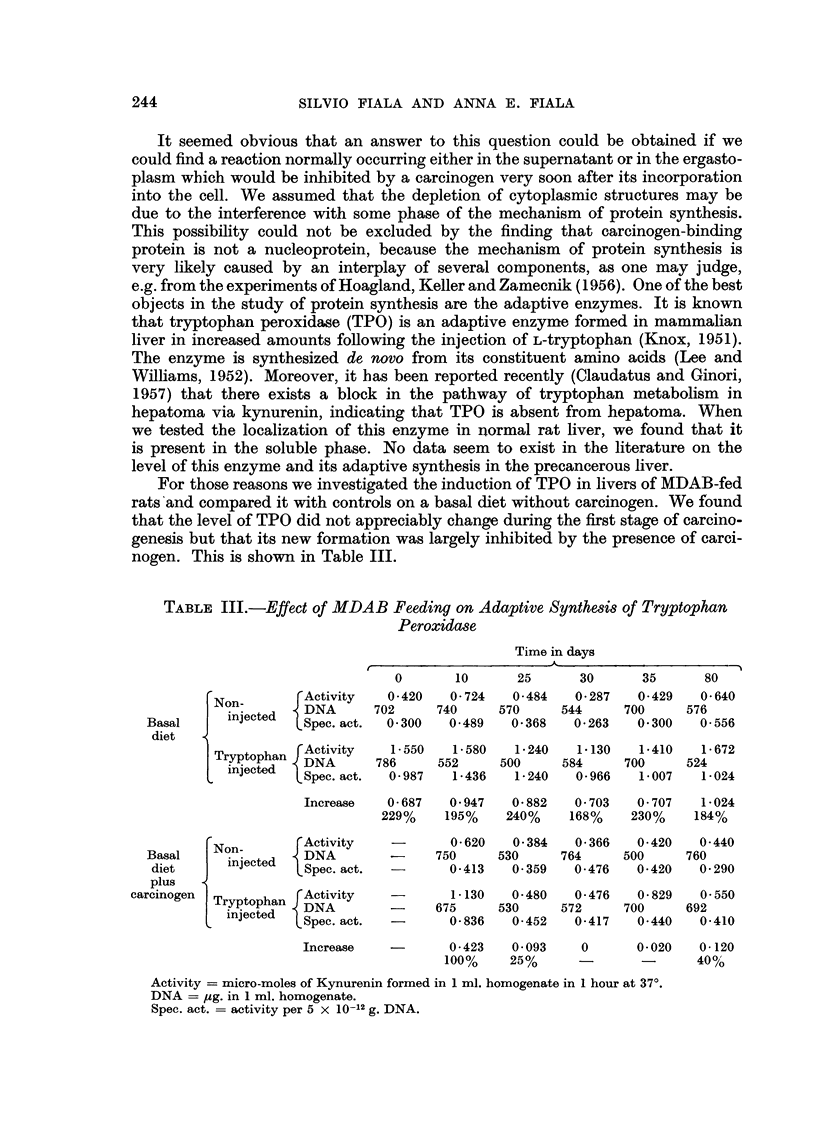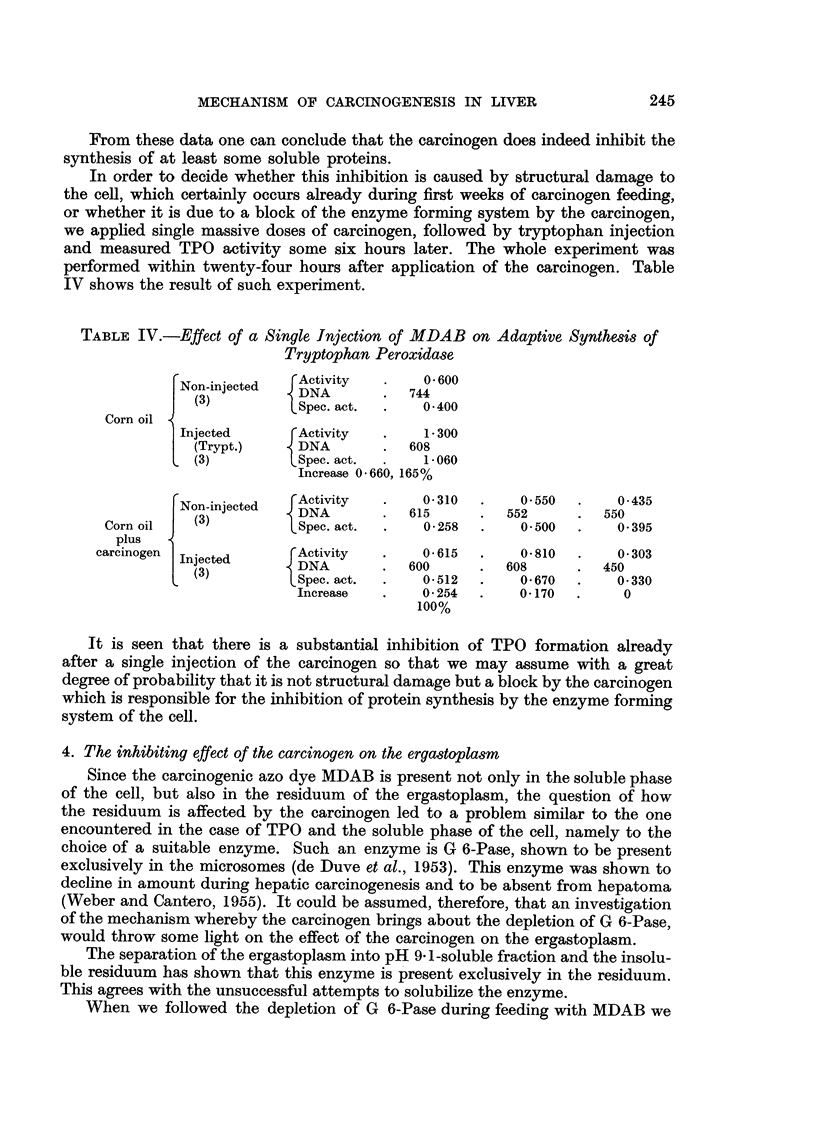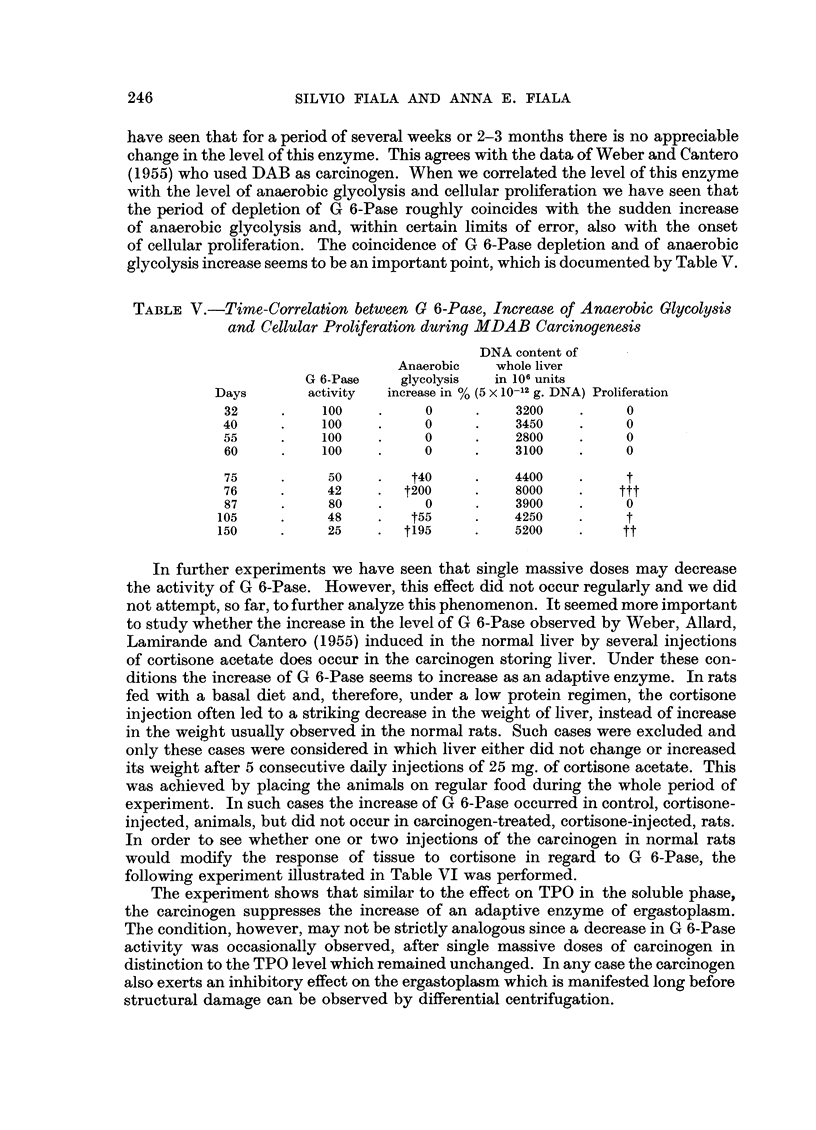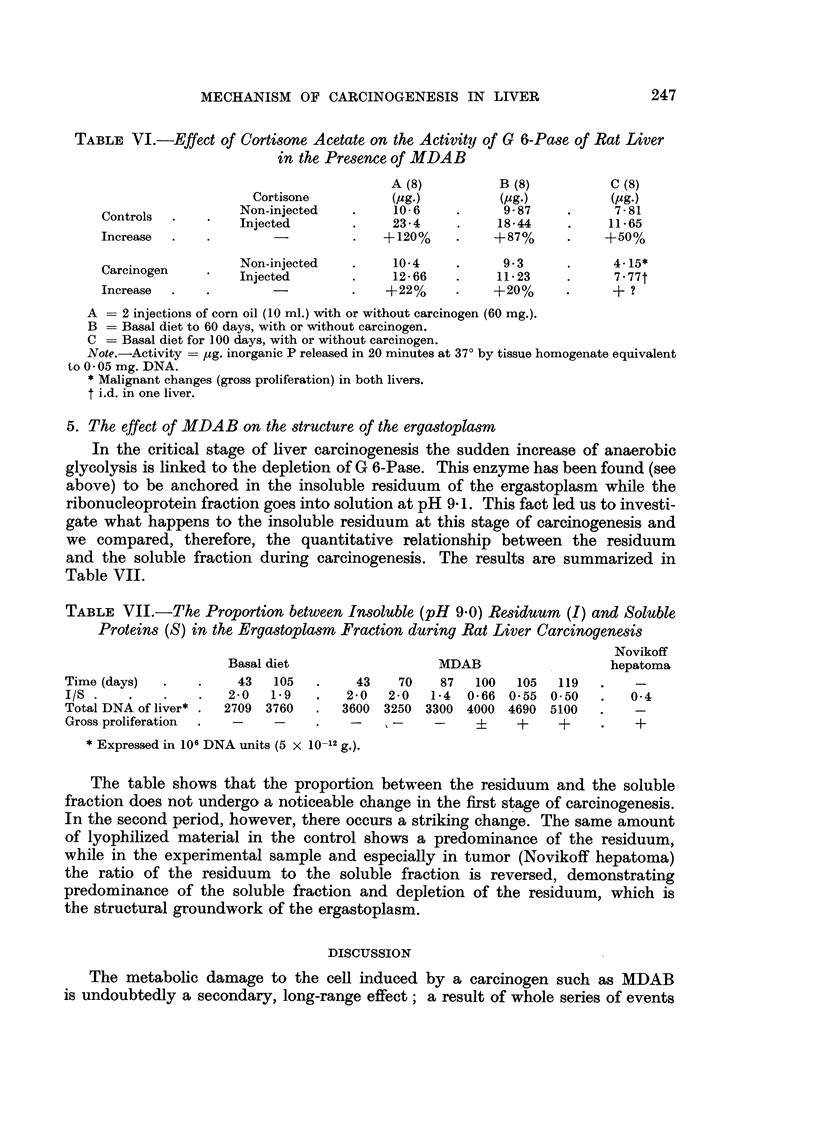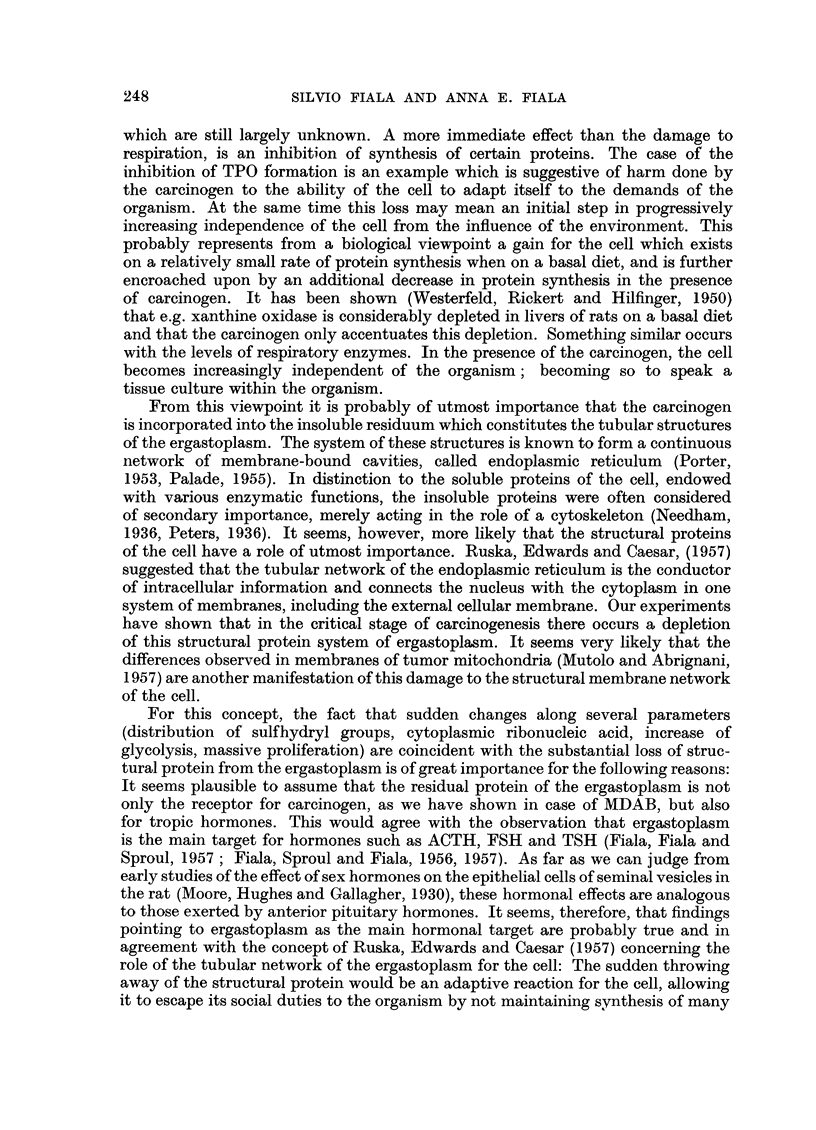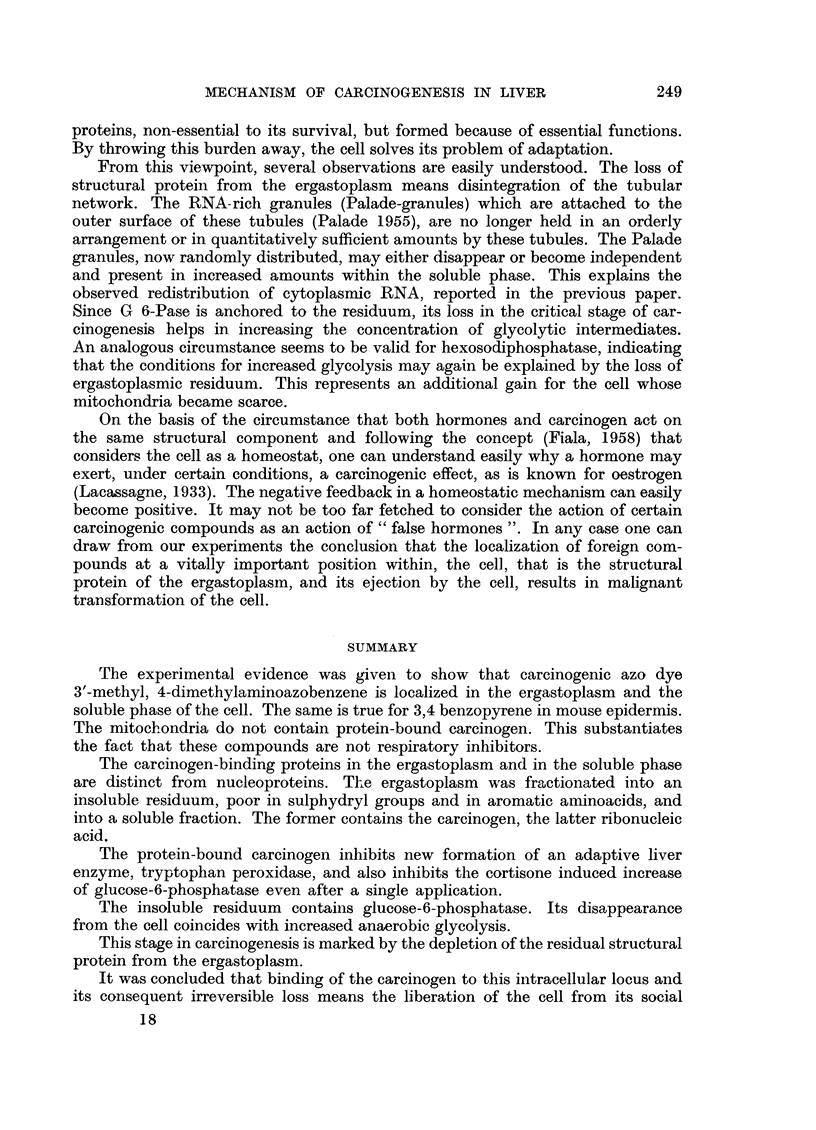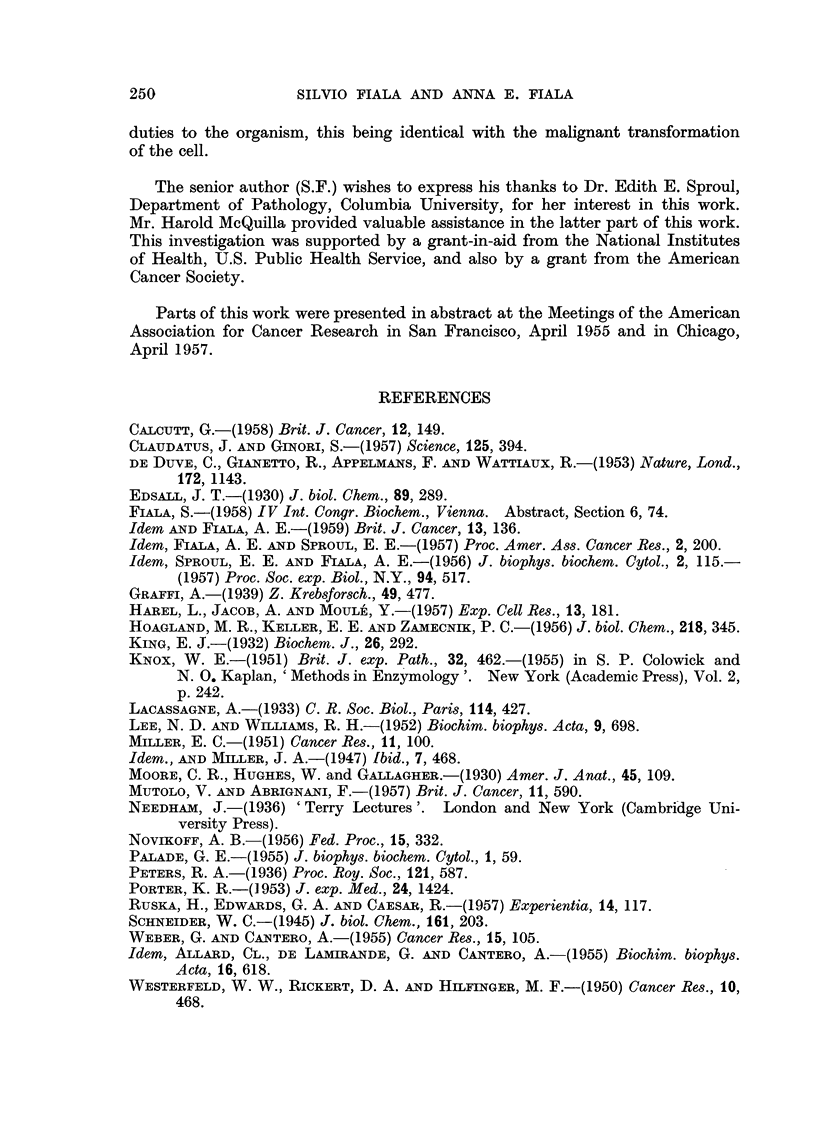# Intracellular Localization of Carcinogen and its Relationship to the Mechanism of Carcinogenesis in Rat Liver

**DOI:** 10.1038/bjc.1959.31

**Published:** 1959-06

**Authors:** Silvio Fiala, Anna E. Fiala


### INTRACELLULAR LOCALIZATION OF CARCINOGEN AND ITS RELATIONSHIP TO THE MECHANISM OF CARCINOGENESIS IN RAT LIVER

SILVIO FIALA AND ANNA E. FIALA

*From the Department of Pathology, Columbia University and Francis Delafield Hospital, New York City*

Received for publication April 13, 1959

THE experimental study of carcinogenesis aims at the finding of the common denominator for metabolic and structural changes which are characteristic of malignant transformation. In the previous paper (Fiala and Fiala, 1959) we tried to throw some light on this problem by correlating in time various changes during the carcinogenic process induced in rat liver by 3′-methyl, 4-dimethylaminoazobenzene. It was found that the whole process consists of two distinct phases. In the first there occurs a depletion of preformed cytoplasmic granules, accompanied by a lowering in the amount of respiratory activity, while glycolysis remains unchanged. Then, some 80 days after initiation of carcinogen feeding, a relatively sudden increase of anaerobic glycolysis takes place, coinciding with or slightly preceding the onset of massive cellular proliferation. At this time there occurs also a marked shift in the distribution of cytoplasmic ribonucleic acid from the ergastoplasm into the supernatant.

It is obvious, therefore, that the single factor we sought for is actually the ratio

$$\frac{\mathrm{N} + \mathrm{S}}{\mathrm{M} + \mathrm{CH} + \mathrm{R}} = \mathrm{K}^*$$

which changes its value in favor of the numerator in that critical period of carcinogenesis which marks the onset of malignant transformation. If we substitute the active masses of subcellular fractions for the respective symbols, the result describes the great preponderance of the nuclei and unorganized soluble phase of the cell over the preformed cytoplasmic elements. This amounts to the lack of cytoplasmic differentiation. If, on the other hand we substitute the corresponding ribonucleic acid for each fraction, we obtain a shift in the distribution of ribonucleic acid from cytoplasmic elements into the nucleus and supernatant. When, finally, we substitute into this ratio the metabolic activities of the corresponding fractions, we obtain a sudden increase in glycolysis (concentrated in the supernatant) over and above the respiration of mitochondria. The positive Warburg quotient

$$\frac{\mathrm{Q}^{\mathrm{O}_2}_{\mathrm{CO}_2}}{\mathrm{Q}_{\mathrm{O}_2}} > 0$$

expressing the appearance of the aerobic glycolysis at this period is only an extreme case of this situation.

* *N* = nucleus.
*M* = mitochondria.
*CH* = " fluffy layer ".
*R* = ergastoplasm.
*S* = soluble fraction.

If the process of carcinogenesis is conceived to be a shift from one equilibrium into another, then one or more factors must exist which keep this equilibrium within normal values. It seems plausible to assume that the protein-bound carcinogen interferes somehow with this regulating activity of the cell. In order to elucidate this fundamental problem of carcinogenesis, it is necessary to establish the localization of the carcinogen within the cell. It seems obvious that any subcellular organelle with a predilection for the carcinogen is most likely its primary target and plays an important role in this regulating activity of the cell. The questions arising next are, therefore, what kind of processes are influenced by the localization of the carcinogen and how is the interference by the carcinogen with the regulating activity of the cell, leading to a new equilibrium at the critical period of carcinogenesis, concerned with the mechanism of this process. These problems outline the program of this paper.

### MATERIALS AND METHODS

Sprague-Dawley rats and, in a few experiments, Swiss albino mice were used as experimental animals. Separation and purification of subcellular fractions was performed as described in the previous paper.

Two criteria were used for estimating the purity of mitochondrial fractions : (a) lack of staining with fresh aqueous solution of Pyronin Y (0·1 per cent) ; (b) the basophilic quotient (B.Q.). The mitochondria apparently do not contain any ribonucleic acid (RNA) at all (Novikoff, 1956) or only small and variable amounts (Harel, Jacob and Moulé, 1957). Their B.Q. (RNA/protein nitrogen) should be, therefore, close to zero. On the other hand the B.Q. of the ergastoplasmic fraction approaches 1. For this reason the B.Q. of the mitochondrial fraction indicates approximately the percentage of contamination with basophilic RNA-rich elements. Thus a B.Q. of 0·5 indicates that the mitochondria are approximately 50 per cent pure, while a B.Q. of 0·2 indicates 80 per cent. purity.

The amount of protein-bound 3′-methyl, 4-dimethylaminoazobenzene (MDAB) was determined spectrophotometrically. The absorption of crystalline MDAB at pH 2·9 (absorption maximum at 520 m$\mu$.) was used as a standard. The protein fraction was hydrolyzed, extracted with ether, the ethereal extracts evaporated to near dryness and residuum redissolved in acid ethanol (Miller and Miller, 1947).

Benzpyrene in 0·5 per cent benzene solution was painted twice on the hairless skin of 1–2 days old mice. The mice were sacrificed 24–48 hours later. The carcinogen was visible by characteristic fluorescence in long UV-light. The mice 1–2 days old were chosen because the skin of older mice is too difficult to homogenize. It is virtually impossible to separate their subcellular fractions because the broken tonofibrils adsorb indiscriminately all cytoplasmic elements. Even in young mice all cytoplasmic elements were heavily contaminated. The advantage of using very young mice for the study of carcinogen localization was confirmed recently by Calcutt (1958). The estimation of contamination of the mitochondrial fraction with ergastoplasm was based on the B.Q. The presence of the carcinogen was determined by measuring the fluorescence of the hydrolyzed protein residue of each fraction, previously precipitated with 5 per cent trichloroacetic acid (TCA) and extracted in a Soxhlet apparatus with boiling ethanol for 48 hours (Miller, 1951). The results were compared with unpainted controls of norma mice of the same litter. Fluorescence was measured using the fluorescent attachlment of a Beckman DU spectrophotometer and taking the fluorescence of 5 mg. of quinine sulfate in 25 ml. water as standard equal to 100 per cent of the total deflection.

For the study of tryptophan peroxidase (TPO) L-tryptophan (78 mg. in 10 ml.) was injected intraperitoneally into half of the animals, while the other half remained uninjected. Six hours after injection all animals were sacrificed. TPO was assayed spectrophotometrically (Knox, 1955) from metaphosphoric acid (5 per cent) extracts of liver samples incubated at 37° for 1 hour and from unincubated controls. The sediments obtained after precipitation with 15 per cent metaphosphoric acid were extracted following Schneider's (1945) procedure and the deoxyribonucleic acid (DNA) was measured by the diphenylamine reaction. In the experiments concerning the effect of the carcinogen on TPO formation, single massive doses of carcinogen (40 mg. of crystalline MDAB dissolved in 10 ml. of corn oil) were injected intraperitoneally into rats kept on a basal diet for four weeks prior to the experiment and a similar number of control animals were injected with corn oil free of carcinogen. Seventeen hours after intraperitoneal injection one half of the control animals and one half of the carcinogen injected animals were injected with tryptophan.

Glucose 6-phosphatase (G 6-Pase) activity was determined by measuring the amount of liberated inorganic phosphate by King's modification of the Fiske-Subba-Row procedure (King, 1932). Homogenates or separated ergastoplasmic fractions were diluted appropriately with Tris-buffer of pH 7·0 and G 6-P (1 ml. of approx. $1 \times 10^{-3}$ M sol.) was added when incubation was started (37°). After 15 minutes the reaction was stopped by adding an equal volume of 10 per cent TCA. In unincubated controls TCA was added before G 6-P. The total volume of mixture was 3·0 ml.

Polarographic determination of protein-bound sulphydryl groups was performed using Brdička ammoniacal solution of cobaltous chloride as indicated in the previous paper.

The proteins of the ergastoplasm were separated into soluble and insoluble fraction by the following procedure : the fraction spun down by 1 hour centrifugation at $59000 \times g$ (Spinco preparative ultracentrifuge) from the homogenate in isotonic sucrose (10 volumes) was resuspended in cold physiological saline and recentrifuged in a Servall SS-1 centrifuge for 2 hours at $30000 \times g$ in the cold room. The supernatant was discarded, the sediment was resuspended in distilled water, dialyzed against distilled water for 48 hours at 4° and lyophilized. The lyophilized material was homogenized at pH 9·2 either in a cold Edsall (1930) solution ($6 \times 10^{-1}$KCl, $1 \times 10^{-2}$M $Na_2CO_3$, $4 \times 10^{-2}$M $NaHCO_3$) or, later, when it was seen that the reproducibility of the results demanded buffered solution, in Tris-buffer pH 9·1. The fraction was left for 1–2 hours in the cold with occasional stirring and then centrifuged for 1 hour at maximum speed in the Servall centrifuge. Both sediment, resuspended in distilled water, and supernatant were dialyzed against large volume of distilled water for 48 hours at 4°. The protein nitrogen of dialyzed subfractions was determined by Nesslerization.

### RESULTS

### 1. *Localization of Carcinogen*

Measurements of isolated subcellular fractions from livers of rats fed with MDAB have shown that purified nuclei contained practically no carcinogen.

The nuclear fraction did not stain pink after precipitation with trichloracetic acid throughout the period of carcinogenesis if basophilic cytoplasmic contaminates were removed by repeated washings with isotonic sucrose. Also the hydrolysis of the purified nuclear fraction did not liberate any carcinogenic azo dye. This demonstrated that no appreciable amount of carcinogen was present in the nuclear fraction. The localization and action of this carcinogen must, therefore, be cytoplasmic.

The presence of the carcinogenic azo dye was noted in all cytoplasmic fractions after precipitation with 5 per cent TCA. The comparisons of amounts of MDAB liberated after hydrolysis of cytoplasmic fractions is shown in Table I. By far the greatest amount of this protein-bound carcinogen appeared in the soluble phase ; considerably less was in the ergastoplasm ; and very little was contained within the mitochondria. Still more significant was the fact that the purification of mitochondria, manifested in lowering of the B.Q. from 0·4 to 0·12 coincident with the removal of contaminating RNA-rich elements, resulted in a proportional loss of protein-bound carcinogen. The carcinogen content of liver mitochondria thus depended on contamination by ergastoplasm.

TABLE I.—*Distribution of MDAB in Cytoplasmic Fractions*

| Fraction | Supernatant | Microsomes | Mitochondria I | Mitochondria II (Purified) |
|---|---|---|---|---|
| Protein nitrogen (mg.) | 19·0 | 14 | 13 | 3 |
| RNA (mg.) | 11·4 | 15·4 | 5·2 | 0·36 |
| B.Q. | 0·6 | 1·1 | 0·4 | 0·12 |
| Dye liberated ($\mu$g.) | 70·0 | 46·0 | 16·0 | 1·1 |
| Specific dye binding | 3·62 | 3·3 | 1·2 | 0·36 |

Fig. 1 shows the absorption spectra of liberated azo dye from separated cytoplasmic fractions. The spectrum calculated for mitochondria with B.Q. approaching zero disappears and is, therefore, drawn as a straight line. Under the idealized conditions of pure mitochondria, no carcinogen would be found.

The slight content of the carcinogen in the mitochondrial fraction dependent on the contamination with ergastoplasm, made clear why this carcinogenic azo dye is not a respiratory inhibitor. This result warranted investigation of the localization of a carcinogenic fluorescent hydrocarbon such as benzopyrene and methylcholanthrene because it was speculated for a long time that mitochondria are the main storage place of these carcinogens. Thus Graffi (1939) treated various cells *in vitro* with benzopyrene and methylcholanthrene dissolved in glycerol and serum and observed by fluorescence their accumulation inside mitochondria stained by Janus Green. The obvious drawbacks of his observation were that he used *in vitro* experiments, which are often less conclusive than those *in vivo*, that he chose for his study cells in which benzopyrene and methylcholanthrene do not produce tumors.

In our experiments we have found that the location of benzopyrene in mouse skin depends on the mode of application. When the carcinogen was applied in lanoline, all cytoplasmic fractions yielded characteristic fluorescence in their alcoholic extracts while no carcinogen seemed to be liberated from protein residua after hydrolysis. On the other hand when the carcinogen was applied in benzene solution, a significant fluorescence could be observed, even after a thorough alcohol extraction, from hydrolyzed protein residua of the supernatant and ergastoplasmic fractions. In the homogenate obtained from 2g. of mouse epidermis, the supernatant was the most strongly fluorescent ; the nuclear fraction was least, although its nitrogen content was relatively the highest. The mitochondrial fraction formed only a comparatively small portion of the protein nitrogen of the homogenate. Its content of fluorescent material was also low after hydrolysis, but, per mg. of nitrogen it was only slightly lower than other cytoplasmic fractions.

FIG. 1.—Protein-bound carcinogen liberated by hydrolysis from separated cytoplasmic fractions of 3 rat livers (43 days of carcinogen feeding). Ethanol-HCl mixture, Vol. 25 ml.

When we obtained in another experiment, a mitochondrial fraction which was less contaminated with RNA-rich elements, no fluorescence was found after hydrolysis.

These results left little doubt that neither MDAB nor 3,4-benzopyrene are stored in mitochondria, which is in conformity with their failure to produce respiratory inhibition. The mitochondria cannot be thought of as targets for any direct action of these carcinogens.

2. *Distribution of MDAB in ribonucleic acid-containing fractions of the cytoplasm*

The presence of protein-bound carcinogen in the supernatant and ergastoplasm, fractions containing ribonucleoproteins, and the absence of the carcinogen in mitochondria, free of RNA in any appreciable amount, necessitated investigation of the relationship of the carcinogen to nucleoproteins. When liver homogenate from rats fed with carcinogenic azo dye (28 days) was centrifuged at 164000 × g (0·88 M sucrose) for 4 hours, about 95 per cent of all ribonucleoprotein material was spun down. The protein-bound azo dye was, nevertheless, present in the supernatant in a greater amount than in all other fractions combined.*

* This experiment was performed (in the Spring of 1955) with the co-operation of Dr. K. McCarty of the Department of Biochemistry, Columbia University.

Attempts were also made to isolate, or at least to concentrate, the carcinogen-binding protein from the soluble phase. The initial steps of separation are shown in Table II. Practically all ribonucleoprotein was precipitated by acidification to pH 5·6. The fraction precipitated by the second addition of ammonium sulfate contained most of the protein-bound carcinogen but gave a completely negative pentose test (Mejbaum's modification of Bial's reaction).

FIG. 2.—Relative proportions of protein nitrogen and protein-bound 3,4-benzpyrene in fractions of mouse epidermis.

TABLE II.—*Initial Steps of Purification of the Protein Binding the Azo Dye*

1. Livers removed separately and homogenized in cold 0·25 M sucrose.
2. Centrifuged, 10 minutes at 13,000 r.p.m. Sediment discarded.
3. Supernatant, 1 M. $CH_3COOH$ added to pH 5·8. Centrifuged, sediment discarded.
4. Supernatant, 1N $NH_4OH$ added to pH 8·0 + $(NH_4)_2SO4$ in powder to concentration 2M. Centrifuged, sediment discarded.
5. Supernatant + 0·1 M. $CH_3COOH$ added to pH 5·4 ; if sediment forms within 10 minutes, centrifuged and sediment discarded.
6. Supernatant + $(NH_4)_2SO_4$ to 3 Moles, centrifuged, reddish supernatant discarded.
7.* Sediment dissolved in

$$\frac{N}{1000} - \frac{N}{100}$$

$NH_4OH$ in a small volume, dialyzed against $H_2O$ 48 hours.

8. Crude fraction lyophilized and stored under vacuum in the cold room.

This lyophilized fraction is the starting point for isolation of carcinogen-binding protein.
* 7 gives a negative pentose test.

The isolated protein fraction had a pale yellow color in solution. Its absorption maximum occurred at 405 mµ. (pH 7·0). The absorption maximum is shifted only slightly toward longer wave-lengths in comparison to the absorption maximum of pure carcinogen dissolved in diluted ethanol and at the same pH (Fig. 3). Normal hydrochloric acid, causing pink coloration, dissolved the protein-dye complex without causing turbidity. It was seen that under these conditions the absorption maximum of the complex was again similar to that of pure carcinogen.

In the ultraviolet region the undenatured protein fraction containing very low absorption at 260 m$\mu$. had an absorption peak at 280 m$\mu$. The negative pentose test and spectra thus presented convincing evidence that carcinogen-binding protein of the supernatant fraction is not a ribonucleoprotein.

In order to establish the relationship of the carcinogen to the ribonucleoproteins in the ergastoplasm, the lyophilized subfractions (pH 9·1 soluble sub-

FIG. 3.—Absorption spectra of carcinogen-binding protein (full line) in ultraviolet and visible region. Spectra of crystalline carcinogen (10 $\mu$g./ml.) at pH 2·9 and 7·0.

fraction and the residuum) were analyzed for ribonucleic acid. It was found that the soluble portion contained practically all the ribonucleoprotein which could be precipitated with $CaCl_2$ and also a protein fraction which did not contain RNA but was relatively rich in polarographically active, protein-bound, sulphydryl groups. The RNA-rich protein fraction could be split off from the soluble subfraction with 1 M NaCl.

The pH 9·1-insoluble residuum dissolved in N/2 NaOH at room temperature only in the course of several hours, giving a yellow solution. Approximately 1/3 of this residuum dissolved in 0·1 M citric acid (pH 2·5), while the addition of 0·1 M Na citrate produced turbidity. In contrast to the soluble fraction, the insoluble residuum was characterized by a low content of polarographically active sulphydryl groups.

The amount of sulphydryl groups in the residuum did not increase after heating to 60° for 15 minutes to any considerable degree. It seemed likely that their presence in the residuum was due to the contamination by the soluble fraction. The residuum differed also from the soluble fraction by a relatively low absorption at 280 m$\mu$. which belongs to aromatic amino acids.

After several hours, the yellow material of the ergastoplasmic residuum dissolved in acidified chloroform. When irradiated in the dark with long ultra-violet light it gave a very intensive green fluorescence. This indicated that the riboflavin (lumiflavin) moiety of yellow enzymes is incorporated into the ergastoplasmic residuum. Ergastoplasmic residuum from tumors such as Novikoff hepatoma did not yield fluorescence under similar conditions. The properties of the residuum and the fact that it contains few if any sulphydryl groups and is very poor in aromatic amino acids, left no doubt that the residuum represents the insoluble, structural, protein framework of the ergastoplasm, while the RNA-rich material represents Palade granules (Palade, 1955) which are attached from the outside to the tubular network (endoplasmic reticulum, Porter, 1953).

Because of the solubility of carcinogen-binding protein in the supernatant, we expected to find carcinogen localized in the soluble subfraction of the ergasto-

FIG. 4.—Comparison of polarographically active free sulphydryl groups in structural (E) and soluble (P) protein fraction of rat liver ergastoplasm.

13 mg. of lyophilized protein fraction dissolved in 5 ml. of 0·5 N NaOH. 0·5 ml. of protein solution added to the mixture of 5 ml. N/10 $NH_4Cl$ in N/10 $NH_4OH$ and 0·5 ml. Co $(NH_3)_6Cl_3$ ($4 \times 10^{-3}$ M).

plasm. Contrary to this expectation the carcinogen was found to be firmly bound mainly to the residuum, apart from ribonucleoproteins, thus providing evidence that it is here where the damaging influence of this carcinogen is primarily localized.

### 3. *The prevention of adaptive formation of tryptophan peroxidase by MDAB*

All changes described so far as induced by carcinogens are long range effects. By the fact that carcinogenic azo dye MDAB is not a respiratory inhibitor and that glycolysis increases only at the onset of malignant transformation (Fiala and Fiala, 1959) the often held assumption that the carcinogen interferes primarily with the metabolism of the cell cannot be held any longer. What then is the primary effect of a carcinogen beyond its binding to one or more chemically, not yet fully characterized proteins ?

It seemed obvious that an answer to this question could be obtained if we could find a reaction normally occurring either in the supernatant or in the ergastoplasm which would be inhibited by a carcinogen very soon after its incorporation into the cell. We assumed that the depletion of cytoplasmic structures may be due to the interference with some phase of the mechanism of protein synthesis. This possibility could not be excluded by the finding that carcinogen-binding protein is not a nucleoprotein, because the mechanism of protein synthesis is very likely caused by an interplay of several components, as one may judge, e.g. from the experiments of Hoagland, Keller and Zamecnik (1956). One of the best objects in the study of protein synthesis are the adaptive enzymes. It is known that tryptophan peroxidase (TPO) is an adaptive enzyme formed in mammalian liver in increased amounts following the injection of L-tryptophan (Knox, 1951). The enzyme is synthesized *de novo* from its constituent amino acids (Lee and Williams, 1952). Moreover, it has been reported recently (Claudatus and Ginori, 1957) that there exists a block in the pathway of tryptophan metabolism in hepatoma via kynurenin, indicating that TPO is absent from hepatoma. When we tested the localization of this enzyme in normal rat liver, we found that it is present in the soluble phase. No data seem to exist in the literature on the level of this enzyme and its adaptive synthesis in the precancerous liver.

For those reasons we investigated the induction of TPO in livers of MDAB-fed rats and compared it with controls on a basal diet without carcinogen. We found that the level of TPO did not appreciably change during the first stage of carcinogenesis but that its new formation was largely inhibited by the presence of carcinogen. This is shown in Table III.

TABLE III.—*Effect of MDAB Feeding on Adaptive Synthesis of Tryptophan Peroxidase*

| | | | Time in days | | | | | |
|---|---|---|---|---|---|---|---|---|
| | | | 0 | 10 | 25 | 30 | 35 | 80 |
| Basal diet | Non-injected | Activity | 0·420 | 0·724 | 0·484 | 0·287 | 0·429 | 0·640 |
| | | DNA | 702 | 740 | 570 | 544 | 700 | 576 |
| | | Spec. act. | 0·300 | 0·489 | 0·368 | 0·263 | 0·300 | 0·556 |
| | Tryptophan injected | Activity | 1·550 | 1·580 | 1·240 | 1·130 | 1·410 | 1·672 |
| | | DNA | 786 | 552 | 500 | 584 | 700 | 524 |
| | | Spec. act. | 0·987 | 1·436 | 1·240 | 0·966 | 1·007 | 1·024 |
| | | Increase | 0·687 | 0·947 | 0·882 | 0·703 | 0·707 | 1·024 |
| | | | 229% | 195% | 240% | 168% | 230% | 184% |
| Basal diet plus carcinogen | Non-injected | Activity | — | 0·620 | 0·384 | 0·366 | 0·420 | 0·440 |
| | | DNA | — | 750 | 530 | 764 | 500 | 760 |
| | | Spec. act. | — | 0·413 | 0·359 | 0·476 | 0·420 | 0·290 |
| | Tryptophan injected | Activity | — | 1·130 | 0·480 | 0·476 | 0·829 | 0·550 |
| | | DNA | — | 675 | 530 | 572 | 700 | 692 |
| | | Spec. act. | — | 0·836 | 0·452 | 0·417 | 0·440 | 0·410 |
| | | Increase | — | 0·423 | 0·093 | 0 | 0·020 | 0·120 |
| | | | | 100% | 25% | — | — | 40% |

Activity = micro-moles of Kynurenin formed in 1 ml. homogenate in 1 hour at 37°.
DNA = $\mu$g. in 1 ml. homogenate.
Spec. act. = activity per $5 \times 10^{-12}$ g. DNA.

From these data one can conclude that the carcinogen does indeed inhibit the synthesis of at least some soluble proteins.

In order to decide whether this inhibition is caused by structural damage to the cell, which certainly occurs already during first weeks of carcinogen feeding, or whether it is due to a block of the enzyme forming system by the carcinogen, we applied single massive doses of carcinogen, followed by tryptophan injection and measured TPO activity some six hours later. The whole experiment was performed within twenty-four hours after application of the carcinogen. Table IV shows the result of such experiment.

TABLE IV.—*Effect of a Single Injection of MDAB on Adaptive Synthesis of Tryptophan Peroxidase*

| | | | | | |
|---|---|---|---|---|---|
| Corn oil | Non-injected (3) | Activity | 0·600 | | |
| | | DNA | 744 | | |
| | | Spec. act. | 0·400 | | |
| | Injected (Trypt.) (3) | Activity | 1·300 | | |
| | | DNA | 608 | | |
| | | Spec. act. | 1·060 | | |
| | | Increase 0·660, 165% | | | |
| Corn oil plus carcinogen | Non-injected (3) | Activity | 0·310 | 0·550 | 0·435 |
| | | DNA | 615 | 552 | 550 |
| | | Spec. act. | 0·258 | 0·500 | 0·395 |
| | Injected (3) | Activity | 0·615 | 0·810 | 0·303 |
| | | DNA | 600 | 608 | 450 |
| | | Spec. act. | 0·512 | 0·670 | 0·330 |
| | | Increase | 0·254 100% | 0·170 | 0 |

It is seen that there is a substantial inhibition of TPO formation already after a single injection of the carcinogen so that we may assume with a great degree of probability that it is not structural damage but a block by the carcinogen which is responsible for the inhibition of protein synthesis by the enzyme forming system of the cell.

### 4. *The inhibiting effect of the carcinogen on the ergastoplasm*

Since the carcinogenic azo dye MDAB is present not only in the soluble phase of the cell, but also in the residuum of the ergastoplasm, the question of how the residuum is affected by the carcinogen led to a problem similar to the one encountered in the case of TPO and the soluble phase of the cell, namely to the choice of a suitable enzyme. Such an enzyme is G 6-Pase, shown to be present exclusively in the microsomes (de Duve *et al.*, 1953). This enzyme was shown to decline in amount during hepatic carcinogenesis and to be absent from hepatoma (Weber and Cantero, 1955). It could be assumed, therefore, that an investigation of the mechanism whereby the carcinogen brings about the depletion of G 6-Pase, would throw some light on the effect of the carcinogen on the ergastoplasm.

The separation of the ergastoplasm into pH 9·1-soluble fraction and the insoluble residuum has shown that this enzyme is present exclusively in the residuum. This agrees with the unsuccessful attempts to solubilize the enzyme.

When we followed the depletion of G 6-Pase during feeding with MDAB we have seen that for a period of several weeks or 2–3 months there is no appreciable change in the level of this enzyme. This agrees with the data of Weber and Cantero (1955) who used DAB as carcinogen. When we correlated the level of this enzyme with the level of anaerobic glycolysis and cellular proliferation we have seen that the period of depletion of G 6-Pase roughly coincides with the sudden increase of anaerobic glycolysis and, within certain limits of error, also with the onset of cellular proliferation. The coincidence of G 6-Pase depletion and of anaerobic glycolysis increase seems to be an important point, which is documented by Table V.

TABLE V.—*Time-Correlation between G 6-Pase, Increase of Anaerobic Glycolysis and Cellular Proliferation during MDAB Carcinogenesis*

| Days | G 6-Pase activity | Anaerobic glycolysis increase in % | DNA content of whole liver in $10^6$ units ($5 \times 10^{-12}$ g. DNA) | Proliferation |
|---|---|---|---|---|
| 32 | 100 | 0 | 3200 | 0 |
| 40 | 100 | 0 | 3450 | 0 |
| 55 | 100 | 0 | 2800 | 0 |
| 60 | 100 | 0 | 3100 | 0 |
| 75 | 50 | †40 | 4400 | † |
| 76 | 42 | †200 | 8000 | ††† |
| 87 | 80 | 0 | 3900 | 0 |
| 105 | 48 | †55 | 4250 | † |
| 150 | 25 | †195 | 5200 | †† |

In further experiments we have seen that single massive doses may decrease the activity of G 6-Pase. However, this effect did not occur regularly and we did not attempt, so far, to further analyze this phenomenon. It seemed more important to study whether the increase in the level of G 6-Pase observed by Weber, Allard, Lamirande and Cantero (1955) induced in the normal liver by several injections of cortisone acetate does occur in the carcinogen storing liver. Under these conditions the increase of G 6-Pase seems to increase as an adaptive enzyme. In rats fed with a basal diet and, therefore, under a low protein regimen, the cortisone injection often led to a striking decrease in the weight of liver, instead of increase in the weight usually observed in the normal rats. Such cases were excluded and only these cases were considered in which liver either did not change or increased its weight after 5 consecutive daily injections of 25 mg. of cortisone acetate. This was achieved by placing the animals on regular food during the whole period of experiment. In such cases the increase of G 6-Pase occurred in control, cortisone-injected, animals, but did not occur in carcinogen-treated, cortisone-injected, rats. In order to see whether one or two injections of the carcinogen in normal rats would modify the response of tissue to cortisone in regard to G 6-Pase, the following experiment illustrated in Table VI was performed.

The experiment shows that similar to the effect on TPO in the soluble phase, the carcinogen suppresses the increase of an adaptive enzyme of ergastoplasm. The condition, however, may not be strictly analogous since a decrease in G 6-Pase activity was occasionally observed, after single massive doses of carcinogen in distinction to the TPO level which remained unchanged. In any case the carcinogen also exerts an inhibitory effect on the ergastoplasm which is manifested long before structural damage can be observed by differential centrifugation.

TABLE VI.—*Effect of Cortisone Acetate on the Activity of G 6-Pase of Rat Liver in the Presence of MDAB*

| | Cortisone | A (8) ($\mu$g.) | B (8) ($\mu$g.) | C (8) ($\mu$g.) |
|---|---|---|---|---|
| Controls | Non-injected | 10·6 | 9·87 | 7·81 |
| | Injected | 23·4 | 18·44 | 11·65 |
| Increase | — | +120% | +87% | +50% |
| Carcinogen | Non-injected | 10·4 | 9·3 | 4·15* |
| | Injected | 12·66 | 11·23 | 7·77† |
| Increase | — | +22% | +20% | + ? |

A = 2 injections of corn oil (10 ml.) with or without carcinogen (60 mg.).
B = Basal diet to 60 days, with or without carcinogen.
C = Basal diet for 100 days, with or without carcinogen.
*Note.*—Activity = $\mu$g. inorganic P released in 20 minutes at 37° by tissue homogenate equivalent to 0·05 mg. DNA.
* Malignant changes (gross proliferation) in both livers.
† i.d. in one liver.

5. *The effect of MDAB on the structure of the ergastoplasm*

In the critical stage of liver carcinogenesis the sudden increase of anaerobic glycolysis is linked to the depletion of G 6-Pase. This enzyme has been found (see above) to be anchored in the insoluble residuum of the ergastoplasm while the ribonucleoprotein fraction goes into solution at pH 9·1. This fact led us to investigate what happens to the insoluble residuum at this stage of carcinogenesis and we compared, therefore, the quantitative relationship between the residuum and the soluble fraction during carcinogenesis. The results are summarized in Table VII.

TABLE VII.—*The Proportion between Insoluble (pH 9·0) Residuum (I) and Soluble Proteins (S) in the Ergastoplasm Fraction during Rat Liver Carcinogenesis*

| | Basal diet | | MDAB | | | | | | Novikoff hepatoma |
|---|---|---|---|---|---|---|---|---|---|
| Time (days) | 43 | 105 | 43 | 70 | 87 | 100 | 105 | 119 | — |
| I/S | 2·0 | 1·9 | 2·0 | 2·0 | 1·4 | 0·66 | 0·55 | 0·50 | 0·4 |
| Total DNA of liver* | 2709 | 3760 | 3600 | 3250 | 3300 | 4000 | 4690 | 5100 | — |
| Gross proliferation | — | — | — | — | — | ± | + | + | + |

* Expressed in $10^6$ DNA units ($5 \times 10^{-12}$ g.).

The table shows that the proportion between the residuum and the soluble fraction does not undergo a noticeable change in the first stage of carcinogenesis. In the second period, however, there occurs a striking change. The same amount of lyophilized material in the control shows a predominance of the residuum, while in the experimental sample and especially in tumor (Novikoff hepatoma) the ratio of the residuum to the soluble fraction is reversed, demonstrating predominance of the soluble fraction and depletion of the residuum, which is the structural groundwork of the ergastoplasm.

DISCUSSION

The metabolic damage to the cell induced by a carcinogen such as MDAB is undoubtedly a secondary, long-range effect; a result of whole series of events which are still largely unknown. A more immediate effect than the damage to respiration, is an inhibition of synthesis of certain proteins. The case of the inhibition of TPO formation is an example which is suggestive of harm done by the carcinogen to the ability of the cell to adapt itself to the demands of the organism. At the same time this loss may mean an initial step in progressively increasing independence of the cell from the influence of the environment. This probably represents from a biological viewpoint a gain for the cell which exists on a relatively small rate of protein synthesis when on a basal diet, and is further encroached upon by an additional decrease in protein synthesis in the presence of carcinogen. It has been shown (Westerfeld, Rickert and Hilfinger, 1950) that e.g. xanthine oxidase is considerably depleted in livers of rats on a basal diet and that the carcinogen only accentuates this depletion. Something similar occurs with the levels of respiratory enzymes. In the presence of the carcinogen, the cell becomes increasingly independent of the organism ; becoming so to speak a tissue culture within the organism.

From this viewpoint it is probably of utmost importance that the carcinogen is incorporated into the insoluble residuum which constitutes the tubular structures of the ergastoplasm. The system of these structures is known to form a continuous network of membrane-bound cavities, called endoplasmic reticulum (Porter, 1953, Palade, 1955). In distinction to the soluble proteins of the cell, endowed with various enzymatic functions, the insoluble proteins were often considered of secondary importance, merely acting in the role of a cytoskeleton (Needham, 1936, Peters, 1936). It seems, however, more likely that the structural proteins of the cell have a role of utmost importance. Ruska, Edwards and Caesar, (1957) suggested that the tubular network of the endoplasmic reticulum is the conductor of intracellular information and connects the nucleus with the cytoplasm in one system of membranes, including the external cellular membrane. Our experiments have shown that in the critical stage of carcinogenesis there occurs a depletion of this structural protein system of ergastoplasm. It seems very likely that the differences observed in membranes of tumor mitochondria (Mutolo and Abrignani, 1957) are another manifestation of this damage to the structural membrane network of the cell.

For this concept, the fact that sudden changes along several parameters (distribution of sulfhydryl groups, cytoplasmic ribonucleic acid, increase of glycolysis, massive proliferation) are coincident with the substantial loss of structural protein from the ergastoplasm is of great importance for the following reasons: It seems plausible to assume that the residual protein of the ergastoplasm is not only the receptor for carcinogen, as we have shown in case of MDAB, but also for tropic hormones. This would agree with the observation that ergastoplasm is the main target for hormones such as ACTH, FSH and TSH (Fiala, Fiala and Sproul, 1957 ; Fiala, Sproul and Fiala, 1956, 1957). As far as we can judge from early studies of the effect of sex hormones on the epithelial cells of seminal vesicles in the rat (Moore, Hughes and Gallagher, 1930), these hormonal effects are analogous to those exerted by anterior pituitary hormones. It seems, therefore, that findings pointing to ergastoplasm as the main hormonal target are probably true and in agreement with the concept of Ruska, Edwards and Caesar (1957) concerning the role of the tubular network of the ergastoplasm for the cell: The sudden throwing away of the structural protein would be an adaptive reaction for the cell, allowing it to escape its social duties to the organism by not maintaining synthesis of many proteins, non-essential to its survival, but formed because of essential functions. By throwing this burden away, the cell solves its problem of adaptation.

From this viewpoint, several observations are easily understood. The loss of structural protein from the ergastoplasm means disintegration of the tubular network. The RNA-rich granules (Palade-granules) which are attached to the outer surface of these tubules (Palade 1955), are no longer held in an orderly arrangement or in quantitatively sufficient amounts by these tubules. The Palade granules, now randomly distributed, may either disappear or become independent and present in increased amounts within the soluble phase. This explains the observed redistribution of cytoplasmic RNA, reported in the previous paper. Since G 6-Pase is anchored to the residuum, its loss in the critical stage of carcinogenesis helps in increasing the concentration of glycolytic intermediates. An analogous circumstance seems to be valid for hexosodiphosphatase, indicating that the conditions for increased glycolysis may again be explained by the loss of ergastoplasmic residuum. This represents an additional gain for the cell whose mitochondria became scarce.

On the basis of the circumstance that both hormones and carcinogen act on the same structural component and following the concept (Fiala, 1958) that considers the cell as a homeostat, one can understand easily why a hormone may exert, under certain conditions, a carcinogenic effect, as is known for oestrogen (Lacassagne, 1933). The negative feedback in a homeostatic mechanism can easily become positive. It may not be too far fetched to consider the action of certain carcinogenic compounds as an action of " false hormones ". In any case one can draw from our experiments the conclusion that the localization of foreign compounds at a vitally important position within, the cell, that is the structural protein of the ergastoplasm, and its ejection by the cell, results in malignant transformation of the cell.

### SUMMARY

The experimental evidence was given to show that carcinogenic azo dye 3′-methyl, 4-dimethylaminoazobenzene is localized in the ergastoplasm and the soluble phase of the cell. The same is true for 3,4 benzopyrene in mouse epidermis. The mitochondria do not contain protein-bound carcinogen. This substantiates the fact that these compounds are not respiratory inhibitors.

The carcinogen-binding proteins in the ergastoplasm and in the soluble phase are distinct from nucleoproteins. The ergastoplasm was fractionated into an insoluble residuum, poor in sulphydryl groups and in aromatic aminoacids, and into a soluble fraction. The former contains the carcinogen, the latter ribonucleic acid.

The protein-bound carcinogen inhibits new formation of an adaptive liver enzyme, tryptophan peroxidase, and also inhibits the cortisone induced increase of glucose-6-phosphatase even after a single application.

The insoluble residuum contains glucose-6-phosphatase. Its disappearance from the cell coincides with increased anaerobic glycolysis.

This stage in carcinogenesis is marked by the depletion of the residual structural protein from the ergastoplasm.

It was concluded that binding of the carcinogen to this intracellular locus and its consequent irreversible loss means the liberation of the cell from its social duties to the organism, this being identical with the malignant transformation of the cell.

The senior author (S.F.) wishes to express his thanks to Dr. Edith E. Sproul, Department of Pathology, Columbia University, for her interest in this work. Mr. Harold McQuilla provided valuable assistance in the latter part of this work. This investigation was supported by a grant-in-aid from the National Institutes of Health, U.S. Public Health Service, and also by a grant from the American Cancer Society.

Parts of this work were presented in abstract at the Meetings of the American Association for Cancer Research in San Francisco, April 1955 and in Chicago, April 1957.

### REFERENCES

CALCUTT, G.—(1958) *Brit. J. Cancer*, **12**, 149.
CLAUDATUS, J. AND GINORI, S.—(1957) *Science*, **125**, 394.
DE DUVE, C., GIANETTO, R., APPELMANS, F. AND WATTIAUX, R.—(1953) *Nature, Lond.*, **172**, 1143.
EDSALL, J. T.—(1930) *J. biol. Chem.*, **89**, 289.
FIALA, S.—(1958) *IV Int. Congr. Biochem., Vienna.* Abstract, Section 6, 74.
*Idem* AND FIALA, A. E.—(1959) *Brit. J. Cancer*, **13**, 136.
*Idem*, FIALA, A. E. AND SPROUL, E. E.—(1957) *Proc. Amer. Ass. Cancer Res.*, **2**, 200.
*Idem*, SPROUL, E. E. AND FIALA, A. E.—(1956) *J. biophys. biochem. Cytol.*, **2**, 115.—(1957) *Proc. Soc. exp. Biol.*, N.Y., **94**, 517.
GRAFFI, A.—(1939) *Z. Krebsforsch.*, **49**, 477.
HAREL, L., JACOB, A. AND MOULÉ, Y.—(1957) *Exp. Cell Res.*, **13**, 181.
HOAGLAND, M. R., KELLER, E. E. AND ZAMECNIK, P. C.—(1956) *J. biol. Chem.*, **218**, 345.
KING, E. J.—(1932) *Biochem. J.*, **26**, 292.
KNOX, W. E.—(1951) *Brit. J. exp. Path.*, **32**, 462.—(1955) in S. P. Colowick and N. O. Kaplan, 'Methods in Enzymology'. New York (Academic Press), Vol. 2, p. 242.
LACASSAGNE, A.—(1933) *C. R. Soc. Biol., Paris*, **114**, 427.
LEE, N. D. AND WILLIAMS, R. H.—(1952) *Biochim. biophys. Acta*, **9**, 698.
MILLER, E. C.—(1951) *Cancer Res.*, **11**, 100.
*Idem.*, AND MILLER, J. A.—(1947) *Ibid.*, **7**, 468.
MOORE, C. R., HUGHES, W. and GALLAGHER.—(1930) *Amer. J. Anat.*, **45**, 109.
MUTOLO, V. AND ABRIGNANI, F.—(1957) *Brit. J. Cancer*, **11**, 590.
NEEDHAM, J.—(1936) 'Terry Lectures'. London and New York (Cambridge University Press).
NOVIKOFF, A. B.—(1956) *Fed. Proc.*, **15**, 332.
PALADE, G. E.—(1955) *J. biophys. biochem. Cytol.*, **1**, 59.
PETERS, R. A.—(1936) *Proc. Roy. Soc.*, **121**, 587.
PORTER, K. R.—(1953) *J. exp. Med.*, **24**, 1424.
RUSKA, H., EDWARDS, G. A. AND CAESAR, R.—(1957) *Experientia*, **14**, 117.
SCHNEIDER, W. C.—(1945) *J. biol. Chem.*, **161**, 203.
WEBER, G. AND CANTERO, A.—(1955) *Cancer Res.*, **15**, 105.
*Idem*, ALLARD, CL., DE LAMIRANDE, G. AND CANTERO, A.—(1955) *Biochim. biophys. Acta*, **16**, 618.
WESTERFELD, W. W., RICKERT, D. A. AND HILFINGER, M. F.—(1950) *Cancer Res.*, **10**, 468.